# Injury and Illness Prevalence and Incidence in Swedish Olympic Athletes: A 3-year Prospective Cohort Study

**DOI:** 10.1186/s40798-026-01035-8

**Published:** 2026-06-03

**Authors:** Kalle Torvaldsson, Sofi Sonesson, Hanna Lindblom, Jörgen Sandberg, Lykke Tamm, Martin Hägglund

**Affiliations:** 1 Department of Health, Medicine and Caring Sciences, Unit of Physiotherapy, Linköping University, Linköping, Sweden; 2 Sport Without Injury ProgrammE (SWIPE), Department of Health, Medicine and Caring Sciences, Linköping University, Linköping, Sweden; 3 Swedish Olympic Committee, Sofiatornet, Olympiastadion, Stockholm, Sweden

## Abstract

**Background:**

Olympic athletes experience a high burden of health problems, and health surveillance is fundamental for understanding and preventing injuries, illnesses, and pain. Most surveillance data on Olympic athletes have focused on the Olympic Games, with limited data on injury, illness, and pain patterns outside the Games period.

**Objectives:**

The primary aim of this study was to describe the prevalence, incidence, burden, and patterns of injury and illness, and pain prevalence, among Swedish Olympic athletes over three consecutive years.

**Methods:**

We conducted prospective weekly health surveillance (April 2022–March 2025) of 225 elite Olympic athletes (51% males; median age 25 years). Health problems were reported using the Oslo Sports Trauma Research Center Questionnaire on Health Problems and diagnosed by the athletes' medical staff.

**Results:**

A total of 1441 new health problems were reported (517 injuries, 924 illnesses), with a weekly prevalence of 23.6% (injuries 15.0%; illnesses 9.1%) and an incidence of 4.6 health problems/athlete/year (injuries 1.5; illnesses 3.1). Athletes lost on average 22 training/competition days annually (injuries 8 days; illnesses 14 days). Injury prevalence was similar between sexes and competitive seasons, with the knee (16%), lumbosacral region (14%), and shoulder (10%) being most affected. Illness prevalence was higher in females than in males (11.4% vs 6.9%; prevalence rate ratio (PRR) 1.6, 95% CI 1.4–2.0) and in winter compared with summer sports (11.4% vs 7.9%; PRR 1.4, 95% CI 1.2–1.7). Respiratory illness comprised 78% of cases. Weekly pain prevalence was 44%, with 65% of pain reports occurring without a concurrent reported health problem.

**Conclusions:**

One in four Swedish Olympic athletes reported a health problem in an average week, with 4.6 new health problems and 22 time-loss days per athlete per year. Injury and illness patterns varied between subgroups, emphasising the need for targeted prevention strategies to reduce injury and illness burden in Olympic athletes.

**Supplementary Information:**

The online version contains supplementary material available at 10.1186/s40798-026-01035-8.

## Key points

- About one in four Swedish Olympic athletes report a health problem each week, and athletes could expect to experience about five health problems and 22 days lost from training and competition per year. Pain is common, with two-thirds reporting weekly pain without reporting any concurrent health problem. Female and winter sport athletes are particularly affected by illness.
- Prevention should focus on respiratory illnesses and injuries to the knee, lower back, shoulder, and ankle. The frequent reporting of pain without a concurrent health problem should be explored further.
- These findings can help National Olympic Committee medical teams target injury and illness prevention and support efforts to optimise medical resources and planning between Olympic Games.

**Supplementary Information:**

The online version contains supplementary material available at 10.1186/s40798-026-01035-8.

## Introduction

Athlete health surveillance is fundamental to understanding the burden of injury and illness in sport, and to develop and assess preventive measures. Injuries and illnesses affect athletes’ health, performance, and success [1–4]. Efforts to safeguard athletes’ health are essential, with effective medical support relying on a comprehensive understanding of injury and illness patterns.

Most health surveillance research on Olympic athletes has focused on the Olympic Games. A recent systematic review and meta-analysis reported incidences of 6.5 injuries and 3.6 illnesses per 1000 athlete-days, with 10% of athletes sustaining an injury and 6% an illness during the Games [5]. These findings provide valuable insights but reflect only a short competition period and may underestimate the true burden of injury and illness. Olympic athletes face diverse physical and psychosocial demands year-round, making multi-year longitudinal surveillance essential to capture the true burden of health problems.

Studies outside the Games have reported a weekly prevalence of health problems of 32% [6], with knee, ankle/foot, lumbosacral, and shoulder injuries, along with respiratory illnesses, accounting for most of the burden [6–8]. Sport-related pain is another important but understudied aspect of athlete health, which may impair performance and well-being; it is a personal, multidimensional experience that may reflect a wide range of phenomena, such as training-related soreness, acute injury, overuse symptoms, or early-stage pathology [9, 10]. The widespread use of analgesics among Olympians and during international competitions suggests that pain is highly prevalent [11–13], yet data on pain prevalence among elite athletes remain largely unknown. Better understanding of pain alongside injury and illness may provide important information for guiding medical support.

## Objectives

The primary aim of this study was to describe the prevalence, incidence, burden, and patterns of injury and illness, and prevalence of pain, among Swedish Olympic athletes during three consecutive years. The secondary aim was to describe and compare the outcomes stratified by sex, competitive season, sport category, and age group.

## Methods

This was a three-year prospective cohort study from April 2022 to March 2025. All athletes were briefed on the study purpose and procedures, advised that participation was voluntary, and gave informed consent. This study was conducted in line with the Declaration of Helsinki and approved by the Swedish Ethical Review Authority (2022-00438-01). This study was reported according to the Strengthening the Reporting of Observational Studies in Epidemiology (STROBE) guideline and the Sport Injury and Illness Surveillance extension, and statistics were checked using the CHecklist for statistical Assessment of Medical Papers (CHAMP) [14–16].

### Settings and Participants

Participants were elite male and female athletes in the Swedish Olympic Committee’s (SOC) Top and Talent programme—an individualised support programme for current and prospective Olympic athletes, primarily in individual sports. Athletes were selected for the programme based on international performance or through a talent selection process for athletes assessed as having the potential to reach elite international level within 3–6 years of programme entry. As part of the programme, athletes received support, including medical, coaching, training, and nutritional services. All athletes included in Top and Talent during the study period were invited to take part in the prospective health surveillance within the SOC and to participate in this study. Athletes could participate in the health surveillance but decline research participation. Athletes who left Top and Talent ended their registration in the study, but data collected until their exit were included. Since a total population was sought, no exclusion criteria were used.

### Data Collection and Definition of Health Problems

Data were collected prospectively from both athletes and SOC or affiliated medical staff (physicians and physiotherapists) using a digital health surveillance application (AthleteMonitoring, FitStats Technologies, Moncton, Canada). Each Sunday, athletes were asked to respond to a weekly questionnaire on health problems (injury/illness) and sports-related pain. Reminders to non-responders were sent out daily by email, SMS, and/or app notifications throughout the week. To enhance questionnaire response rates, athlete support staff received regular summaries of completion status to facilitate follow-up with non-responding athletes.

Health problems were primarily self-reported by athletes using the Oslo Sports Trauma Research Center (OSTRC) Questionnaire on Health Problems (OSTRC-H2) [17]. OSTRC-H2 includes four questions regarding the consequences of health problems on participation, modified training/competition, performance, and symptoms, considering the preceding seven days. To capture the prevalence of pain, athletes responded to question four in the OSTRC Overuse Injury Questionnaire (OSTRC-O2) (Table 1), irrespective whether they reported a health problem or not [17].

**Table 1:** Definitions

Health problem	Any injury or illness an athlete considered to be a reduction in their normal state of full health, irrespective of its consequences on sports participation or performance, or whether the athlete sought medical attention [17]
Substantial health problem	Injury/illness resulting in a moderate or severe reduction in training volume or sports performance or a complete inability to participate in sport [18]
Injury	Physical damage or notable injury on body tissue, irrespective of the need for medical attention or consequences on participation [16]
Gradual-onset injury	An injury without a clearly identifiable sudden event at onset [16]
Sudden-onset injury	An injury caused by a specific identifiable event [16]
Illness	Complaint or symptom, not related to injury, irrespective of the need for medical attention or consequences on participation. Illnesses include both physical and psychological illness [16]
Pain	Defined based on question four of the OSTRC-O2 (‘To what extent have you experienced pain related to your sport during the past 7 days?’) and response options ‘No pain’, ‘Mild pain’, ‘Moderate pain’, or ‘Severe pain’ [17]
Weekly prevalence	Number of weekly questionnaires with a reported health problem (new or recurring) or pain, divided by the total number of weekly questionnaires. Presented as the proportion of athletes affected by a health problem or pain in an average week
Incidence	Number of new health problems divided by the total number of athlete-days and multiplied by 365 days. Presented as the number of health problems per athlete per year
Severity	Proportion of total time-loss (days) attributable to each injury location, injury type, onset, and illness organ system Cumulative severity score for each health problem aggregated over time based on the OSTRC-H2 weekly severity score (0–100). Presented as a mean cumulative severity score [17]
Burden	Number of training/competition days lost divided by the total number of athlete-days multiplied by 365 days. Expressed as the number of time-loss days due to health problems per athlete per year
Training/competition availability	Sum of days available to train and compete divided by the sum of potential participation days. Expressed as the average percentage of availability over the study period
Cyclic sports	Sports characterised by repetitive, continuous movement patterns and high-volume loads, demanding high aerobic capacity and muscular endurance (e.g. cross-country skiing, rowing, speed skating and triathlon)
Full-contact sports	Sports characterised by direct physical contact and high-energy impacts between athletes, demanding rapid force production and absorption (e.g. boxing and wrestling)
High-impact sports	Sports characterised by explosive movements, high ground reaction forces, or high-velocity impacts during landing, jumping, or acceleration/deceleration (e.g. diving, freestyle skiing, gymnastics and snowboard)
Precision sports	Sports characterised by prolonged static positions or repetitive precision movements (e.g. curling and shooting)
Reactive sports	Sports characterised by multidirectional and unpredictable reactive movements with frequent changes of direction and rapid acceleration and deceleration (e.g. badminton, beach volleyball and fencing)

*OSTRC-O2* Oslo Sports Trauma Research Center Overuse Injury Questionnaire, *OSTRC-H2* Oslo Sports Trauma Research Center Questionnaire on Health Problems

Athletes who reported a health problem in the weekly questionnaire gave additional information about, for example, the type of health problem, injury location, illness symptoms, and number of time-loss days the preceding week. Athletes could report multiple health problems each week, and, when they did so, were instructed to start with the most severe problem. Athletes and medical staff were also able to report health problems separately from the weekly questionnaire [accounting for 3% (*n* = 40) of all reported health problems]. Medical staff were notified when an athlete reported a health problem and followed up to validate the report, correct any erroneous information, and register a diagnosis using the Orchard Sports Injury and Illness Classification System (OSIICS 13.5). Injuries were considered to have a non-specific diagnosis when the body part or tissue type was unspecified (Z in the first or second letter of the OSIICS code), and for illnesses when the organ system or aetiology was unspecified (Z in the second or third letter). All health problems were reviewed for accuracy and completeness by the authors, and any inconsistencies or missing information were addressed in collaboration with SOC medical staff. Health problems, injuries, and illnesses were defined according to OSTRC-H2 and based on the IOC consensus statement on reporting data on injury and illness in sports (Table 1) [16, 17].

### Sport Category Grouping

Sports were categorised into five categories (cyclic, full-contact, high-impact, precision, and reactive sports), as used by the SOC high performance team, to facilitate meaningful implementation within the SOC (Table 1 and Online Resource 1).

### Statistical Analysis

Health problems that existed at baseline [*n* = 87 (68 injuries, 19 illnesses)] or were not related to training/competition (*n* = 20 injuries) were excluded from incidence calculations but included in prevalence calculations. Descriptive data were summarised using mean, median, frequencies, interquartile ranges (IQR), and 95% confidence intervals (CI), as appropriate. Injury and illness weekly prevalence, annual incidence, severity, burden, training/competition availability, and pain prevalence were calculated and analysed for the total population and subgroups (when ≥ 8 athletes were available) based on sex (female, male), competitive season (summer, winter sports), sport category, and age group (Table 1). Age was defined as the athlete’s age in the calendar year of data collection, allowing contribution to multiple age groups over the study period. Burden of the most prevalent injury locations and illness organ systems was visualised in a risk matrix, with weekly prevalence plotted against mean cumulative severity score. Unadjusted prevalence and incidence rates were calculated using generalised linear models with a Poisson distribution and log link, including the natural logarithm of exposure (athlete-weeks for prevalence, athlete-years for incidence) as an offset variable. CIs were adjusted for potential overdispersion with the Pearson scale parameter. Rates were estimated and reported based on observed time at risk. Prevalence rate ratios (PRR) and incidence rate ratios (IRR) were calculated to compare outcomes by sex and competitive season. Data were not imputed for non-response weeks, and event counts and days lost were not extrapolated beyond the observed weeks. Analyses were performed using IBM SPSS Statistics for Windows, version 29 (IBM Corp., Armonk, N.Y., USA).

## Results

Among 239 athletes included in Top and Talent across three years, eight declined research participation and an additional six did not partake in health surveillance. Thus, 225 athletes (111 females, 114 males) from 35 sports were included (Online Resource 1), contributing 15142 athlete-weeks (females 7568, males 7574). Median age at inclusion was 25 years [IQR 22–29; female 25 years (IQR 22–29), males 25 years (IQR 22–28)]. Median observation period per athlete was 95 weeks (IQR 36–157), with 77 athletes (34%) followed for the entire study period. The overall response rate for the weekly health surveillance was 73% (median 81%; IQR 55–98%).

In total, 202 athletes reported 1441 health problems, including 517 injuries (2267 injury weeks; females 1113, males 1154) and 924 illnesses (1385 illness weeks; females 862, males 523). All health problems received a diagnosis code, of which 165 (11%) were non-specific.

### Prevalence, Incidence, and Burden of Health Problems

**Fig. 1:**
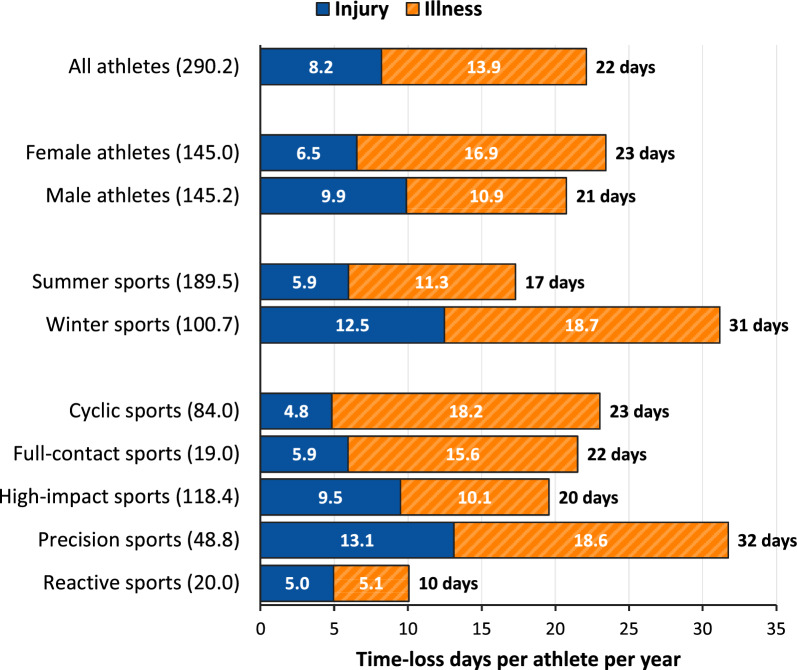
Burden of injury and illness by athlete subgroup. Numbers in brackets represent the total number of athlete-years

The weekly prevalence of any health problem was 23.6% (95% CI 21.6%–25.7%), and of substantial health problems 13.1% (95% CI 11.9%–14.5%). The incidence of new health problems was 4.6 (95% CI 4.3–4.9) per athlete per year. Females reported higher prevalence (25.5% vs 21.6%; PRR 1.18, 95% CI 1.0–1.4) and incidence (5.4 vs 3.8; IRR 1.43, 95% CI 1.2–1.7) than males (Table 2). On average, athletes lost 22 days (95% CI 20–25) of training and competition annually due to health problems (females 23 days, 95% CI 21–27; males 21 days, 95% CI 17–25), corresponding to an overall availability of 94% (Fig. 1).

**Table 2:** Weekly prevalence and annual incidence of any and substantial health problems for subgroups, for the total cohort and stratified by sex

	Weekly prevalence (%, 95% CI)			Incidence (cases/athlete/year, 95% CI)		
	Total	Female	Male	Total	Female	Male
Health problems						
Any health problem	23.6 (21.6–25.7)	25.5 (23.0–28.3)	21.6 (18.7–24.9)	4.6 (4.3–4.9)	5.4 (4.9–5.9)	3.8 (3.4–4.3)
Substantial health problems^a^	13.1 (11.9–14.5)	14.1 (12.4–16.0)	12.1 (10.3–14.3)	2.9 (2.7–3.2)	3.3 (3.0–3.7)	2.5 (2.2–2.9)
Time-loss health problems	13.0 (11.9–14.2)	14.4 (13.0–16.0)	11.6 (10.0–13.5)	3.5 (3.3–3.8)	4.1 (3.7–4.5)	2.9 (2.6–3.3)
Summer sports	21.9 (19.5–24.5)	24.7 (21.3–28.6)	19.0 (15.9–22.8)	4.3 (3.9–4.8)	4.9 (4.3–5.5)	3.8 (3.2–4.4)
Winter sports	26.7 (23.5–30.4)	27.1 (23.7–30.9)	26.4 (21.0–33.1)	5.1 (4.5–5.7)	6.4 (5.6–7.3)	3.8 (3.1–4.6)
Sport category						
Cyclic sports	22.1 (19.3–25.4)	24.3 (20.4–28.9)	19.7 (15.8–24.6)	5.6 (5.0–6.2)	6.2 (5.5–7.1)	4.8 (4.0–5.8)
Full-contact sports	31.7 (24.6–40.8)	37.3 (27.4–50.7)	24.3 (15.9–37.2)	6.8 (5.4–8.5)	7.0 (5.4–9.1)	6.5 (4.2–9.9)
High-impact sports	22.7 (19.5–26.6)	21.0 (17.1–25.8)	24.6 (19.5–30.9)	3.7 (3.2–4.2)	3.8 (3.2–4.5)	3.6 (3.0–4.3)
Precision sports	27.7 (23.0–33.2)	31.8 (27.1–37.3)	22.9 (15.4–34.2)	5.3 (4.5–6.3)	7.4 (6.3–8.7)	2.9 (2.1–4.1)
Reactive sports	16.5 (10.0–27.1)	NA	12.7 (6.9–23.4)	2.1 (1.5–2.8)	NA	1.9 (1.3–2.8)
Injuries						
All injuries	15.0 (13.1–17.1)	14.7 (12.4–17.5)	15.2 (12.5–18.6)	1.5 (1.3–1.7)	1.6 (1.4–1.9)	1.4 (1.2–1.7)
Substantial injuries^a^	7.0 (5.9–8.4)	6.7 (5.2–8.5)	7.4 (5.8–9.5)	0.8 (0.7–0.9)	0.8 (0.6–0.9)	0.8 (0.6–1.0)
Time-loss injuries	5.6 (4.6–6.6)	5.1 (4.1–6.4)	6.0 (4.6–7.8)	0.8 (0.7–0.9)	0.8 (0.6–1.0)	0.8 (0.7–1.0)
Summer sports	14.3 (12.2–16.9)	15.9 (12.7–19.8)	12.8 (10.0–16.4)	1.6 (1.3–1.8)	1.6 (1.3–1.9)	1.5 (1.2–1.9)
Winter sports	16.2 (13.0–20.1)	12.5 (9.7–16.2)	19.8 (14.4–27.1)	1.4 (1.2–1.8)	1.6 (1.2–2.0)	1.3 (0.9–1.8)
Sport category						
Cyclic sports	11.0 (8.7–14.0)	11.9 (8.7–16.3)	10.1 (7.2–14.4)	1.6 (1.3–1.9)	1.7 (1.3–2.2)	1.4 (1.0–2.0)
Full-contact sports	21.4 (15.0–30.6)	27.2 (17.4–42.5)	13.8 (8.3–22.8)	2.7 (2.0–3.6)	2.6 (1.9–3.5)	2.8 (1.7–4.8)
High-impact sports	16.7 (13.5–20.6)	14.5 (10.8–19.4)	19.1 (14.2–25.7)	1.5 (1.2–1.8)	1.4 (1.1–1.9)	1.5 (1.1–2.0)
Precision sports	15.4 (11.2–21.2)	12.8 (9.0–18.1)	18.4 (11.1–30.5)	1.3 (0.9–1.7)	1.4 (0.9–2.0)	1.2 (0.7–1.9)
Reactive sports	14.0 (7.8–25.2)	NA	10.6 (5.2–21.8)	1.0 (0.6–1.7)	NA	0.9 (0.5–1.7)
Illnesses						
All illnesses	9.1 (8.3–10.0)	11.4 (10.2–12.7)	6.9 (5.9–8.0)	3.1 (2.9–3.4)	3.9 (3.5–4.3)	2.4 (2.1–2.7)
Substantial illnesses^a^	6.3 (5.7–7.0)	7.6 (6.7–8.6)	5.0 (4.3–5.9)	2.2 (2.0–2.4)	2.6 (2.3–2.9)	1.8 (1.5–2.0)
Time-loss illnesses	7.7 (7.0–8.4)	9.5 (8.5–10.6)	5.9 (5.1–6.8)	2.7 (2.5–3.0)	3.3 (3.0–3.7)	2.1 (1.9–2.5)
Summer sports	7.9 (7.0–8.9)	9.3 (8.0–10.8)	6.6 (5.4–8.0)	2.8 (2.5–3.1)	3.3 (2.9–3.8)	2.3 (1.9–2.7)
Winter sports	11.4 (9.9–13.1)	15.3 (13.1–17.8)	7.6 (5.9–9.7)	3.7 (3.3–4.2)	4.9 (4.2–5.6)	2.6 (2.0–3.2)
Sport category						
Cyclic sports	11.5 (10.1–13.1)	12.9 (10.9–15.2)	10.1 (8.2–12.3)	4.0 (3.6–4.5)	4.6 (4.0–5.3)	3.4 (2.8–4.2)
Full-contact sports	11.4 (8.0–16.1)	11.7 (7.8–17.4)	11.0 (5.9–20.4)	4.1 (3.1–5.5)	4.5 (3.2–6.4)	3.7 (2.3–5.8)
High-impact sports	6.3 (5.3–7.4)	6.9 (5.6–8.4)	5.7 (4.4–7.5)	2.2 (1.9–2.6)	2.4 (1.9–2.9)	2.1 (1.7–2.7)
Precision sports	13.8 (11.4–16.7)	20.1 (17.0–23.9)	6.5 (4.2–9.8)	4.1 (3.4–5.0)	6.1 (5.1–7.2)	1.8 (1.3–2.6)
Reactive sports	2.5 (1.6–3.8)	NA	2.1 (1.3–3.3)	1.1 (0.7–1.7)	NA	1.0 (0.6–1.7)

*CI* confidence interval, *NA* not applicable (e.g. due to no injury/illness cases or small sample)

^a^Substantial injuries/illnesses are defined as those that resulted in a moderate or severe reduction in training volume or sports performance or a complete inability to participate in sport

### Injury Prevalence, Incidence, and Burden

The weekly prevalence of all injuries was 15.0% (95% CI 13.1%–17.1%), and of substantial injuries 7.0% (95% CI 5.9%–8.4%). The incidence was 1.5 (95% CI 1.3–1.7) injuries per athlete per year, and athletes lost on average eight days (95% CI 6–10) annually due to injury (females 7 days, 95% CI 5–9; males 10 days, 95% CI 7–14). Overall injury prevalence and incidence were similar between sexes (PRR 1.04, 95% CI 0.8–1.3; IRR 0.91, 95% CI 0.7–1.2) and competitive seasons (PRR 0.89, 95% CI 0.7–1.2; IRR 1.08, 95% CI 0.8–1.4) (Table 2). Among females, prevalence was highest in summer and in full-contact sports, while among males it was highest in winter and in high-impact sports (data for sport categories are presented in Online Resource 2). There was a tendency towards higher injury prevalence among younger athletes (Online Resource 3). Injury prevalence and incidence for subgroups are presented in Table 2, and for injury locations, types, and onset in Online Resource 4.

### Injury Characteristics

**Fig. 2:**
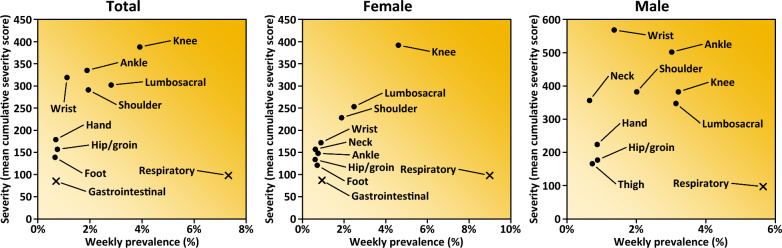
Risk matrices illustrating the relationship between weekly prevalence (%) and severity (mean cumulative severity score) for the ten injury locations/illness organ systems with the highest prevalence, for the total cohort and stratified by sex. Round markers refer to injury and cross markers to illness

**Fig. 3:**
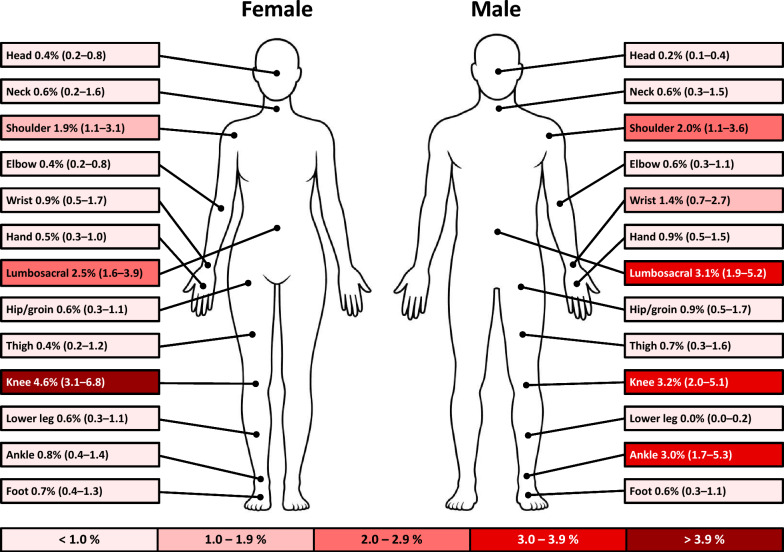
Weekly prevalence (%, 95% CI) stratified by injury location and sex (upper arm, forearm, chest, thoracic spine and abdomen not included due to low overall prevalence)

The most common injury locations were the knee (16%), lumbosacral region (14%), shoulder (10%), and ankle (7%), which also accounted for the greatest injury burden (Figs. 2 and 3). The non-specific category comprised 37% of all injuries, while muscle injuries (14%), joint sprains (11%), and tendinopathies (10%) were the most frequent specified types (Table 3).

**Table 3:** Number of injuries (% of total number of injuries) and time-loss days (% of total time-loss due to injury) by injury location, type, and onset, for the total cohort and stratified by sex

	Number of injuries (% of total)			Time–loss days (% of total)		
	Total (*N* = 517)	Female (*N* = 279)	Male (*N* = 238)	Total (*N* = 2382)	Female (*N* = 945)	Male (*N* = 1437)
Injury location						
Head and neck	44 (9%)	27 (10%)	17 (7%)	179 (8%)	109 (12%)	70 (5%)
Head	22 (4%)	11 (4%)	11 (5%)	83 (3%)	45 (5%)	38 (3%)
Neck	22 (4%)	16 (6%)	6 (3%)	96 (4%)	64 (7%)	32 (2%)
Upper limb	130 (25%)	71 (25%)	59 (25%)	563 (24%)	201 (21%)	362 (25%)
Shoulder	54 (10%)	32 (11%)	22 (9%)	316 (13%)	142 (15%)	174 (12%)
Upper arm	2 (< 1%)	2 (1%)	0 (–)	2 (< 1%)	2 (< 1%)	0 (–)
Elbow	18 (3%)	7 (3%)	11 (5%)	24 (1%)	6 (1%)	18 (1%)
Forearm	4 (1%)	2 (1%)	2 (1%)	8 (< 1%)	7 (1%)	1 (< 1%)
Wrist	27 (5%)	17 (6%)	10 (4%)	167 (7%)	34 (4%)	133 (9%)
Hand	25 (5%)	11 (4%)	14 (6%)	46 (2%)	10 (1%)	36 (3%)
Trunk	108 (21%)	52 (19%)	56 (24%)	461 (19%)	169 (18%)	292 (20%)
Chest	12 (2%)	7 (3%)	5 (2%)	23 (1%)	3 (< 1%)	20 (1%)
Thoracic spine	12 (2%)	8 (3%)	4 (2%)	16 (1%)	6 (1%)	10 (1%)
Lumbosacral	71 (14%)	34 (12%)	37 (16%)	388 (16%)	159 (17%)	229 (16%)
Abdomen	13 (3%)	3 (1%)	10 (4%)	34 (1%)	1 (< 1%)	33 (2%)
Lower limb	219 (42%)	120 (43%)	99 (42%)	969 (41%)	409 (43%)	560 (39%)
Hip/groin	30 (6%)	14 (5%)	16 (7%)	109 (5%)	28 (3%)	81 (6%)
Thigh	23 (4%)	10 (4%)	13 (5%)	47 (2%)	19 (2%)	28 (2%)
Knee	82 (16%)	46 (16%)	36 (15%)	550 (23%)	254 (27%)	296 (21%)
Lower leg	18 (3%)	15 (5%)	3 (1%)	43 (2%)	39 (4%)	4 (< 1%)
Ankle	34 (7%)	16 (6%)	18 (8%)	133 (6%)	37 (4%)	96 (7%)
Foot	32 (6%)	19 (7%)	13 (5%)	87 (4%)	32 (3%)	55 (4%)
Multiple regions	16 (3%)	9 (3%)	7 (3%)	210 (9%)	57 (6%)	153 (11%)
Injury type						
Muscle/tendon	146 (28%)	82 (29%)	64 (27%)	517 (22%)	236 (25%)	281 (20%)
Muscle injury	71 (14%)	39 (14%)	32 (13%)	214 (9%)	120 (13%)	94 (7%)
Muscle contusion	19 (4%)	13 (5%)	6 (3%)	42 (2%)	28 (3%)	14 (1%)
Muscle compartment syndrome	3 (1%)	1 (< 1%)	2 (1%)	2 (< 1%)	2 (< 1%)	0 (–)
Tendinopathy	50 (10%)	27 (10%)	23 (10%)	153 (6%)	81 (9%)	72 (5%)
Tendon rupture	3 (1%)	2 (1%)	1 (< 1%)	106 (4%)	5 (1%)	101 (7%)
Nervous	9 (2%)	4 (1%)	5 (2%)	191 (8%)	51 (5%)	140 (10%)
Brain/spinal cord injury/peripheral nerve injury	9 (2%)	4 (1%)	5 (2%)	191 (8%)	51 (5%)	140 (10%)
Bone	35 (7%)	13 (5%)	22 (9%)	218 (9%)	40 (4%)	178 (12%)
Fracture	19 (4%)	4 (1%)	15 (6%)	184 (8%)	31 (3%)	153 (11%)
Bone stress injury	5 (1%)	2 (1%)	3 (1%)	19 (1%)	7 (1%)	12 (1%)
Bone contusion	11 (2%)	7 (3%)	4 (2%)	15 (1%)	2 (< 1%)	13 (1%)
Cartilage/synovium/bursa	43 (8%)	21 (8%)	22 (9%)	407 (17%)	74 (8%)	333 (23%)
Cartilage	26 (5%)	15 (5%)	11 (5%)	270 (11%)	66 (7%)	204 (14%)
Arthritis	4 (1%)	3 (1%)	1 (< 1%)	4 (< 1%)	4 (< 1%)	0 (–)
Synovitis/capsulitis	5 (1%)	2 (1%)	3 (1%)	10 (< 1%)	4 (< 1%)	6 (< 1%)
Bursitis	8 (2%)	1 (< 1%)	7 (3%)	123 (5%)	0 (–)	123 (9%)
Ligament/joint capsule	64 (12%)	41 (15%)	23 (10%)	386 (16%)	240 (25%)	146 (10%)
Joint sprain (ligament tear or acute instability episode)	59 (11%)	36 (13%)	23 (10%)	327 (14%)	181 (19%)	146 (10%)
Chronic instability	5 (1%)	5 (2%)	0 (–)	59 (2%)	59 (6%)	0 (–)
Superficial tissues/skin/vessels	27 (5%)	16 (6%)	11 (5%)	74 (3%)	52 (6%)	22 (2%)
Contusion (superficial)/vascular trauma	16 (3%)	10 (4%)	6 (3%)	35 (1%)	26 (3%)	9 (1%)
Laceration	9 (2%)	6 (2%)	3 (1%)	39 (2%)	26 (3%)	13 (1%)
Abrasion	2 (< 1%)	0 (–)	2 (1%)	0 (–)	0 (–)	0 (–)
Non-specific	193 (37%)	102 (37%)	91 (38%)	589 (25%)	252 (27%)	337 (23%)
Injury onset						
Gradual onset	267 (52%)	141 (51%)	126 (53%)	895 (38%)	394 (42%)	501 (35%)
Sudden onset	250 (48%)	138 (49%)	112 (47%)	1487 (62%)	551 (58%)	936 (65%)

### Illness Prevalence, Incidence, and Burden

The weekly prevalence of all illnesses was 9.1% (95% CI 8.3%–10.0%), and of substantial illnesses 6.3% (95% CI 5.7%–7.0%). The incidence was 3.1 (95% CI 2.9–3.4) illnesses per athlete per year. Athletes lost on average 14 days (95% CI 13–15) annually due to illness (females 17 days, 95% CI 15–19; males 11 days, 95% CI 9–13). The prevalence and incidence of illness were higher in females than in males (PRR 1.65, 95% CI 1.4–2.0; IRR 1.62, 95% CI 1.4–1.9), and in winter than in summer sports (PRR 1.44, 95% CI 1.2–1.7; IRR 1.33, 95% CI 1.1–1.6) (Table 2). Among females, illness prevalence was highest in precision sports, and among males in full-contact sports (data for sport categories are presented in Online Resource 2). Illness prevalence and incidence were generally lower among younger athletes (Online Resource 3). Illness prevalence and incidence for subgroups are presented in Table 2, and for organ systems in Online Resource 5.

### Illness Characteristics

Respiratory illnesses were most common (78% of all illnesses) and contributed to the greatest illness-related burden (Fig. 2). Illnesses by organ systems are presented in Table 4.

**Table 4:** Number of illnesses (% of total number of illnesses) and time-loss days (% of total time-loss due to illness) by organ system/region, for the total cohort and stratified by sex

	Number of illnesses (% of total)			Time–loss days (% of total)		
	Total (*N* = 924)	Female (*N* = 571)	Male (*N* = 353)	Total (*N* = 4034)	Female (*N* = 2455)	Male (*N* = 1579)
Organ system/region						
Cardiovascular	1 (< 1%)	0 (–)	1 (< 1%)	2 (< 1%)	0 (–)	2 (< 1%)
Dermatological	19 (2%)	14 (2%)	5 (1%)	80 (2%)	60 (2%)	17 (1%)
Dental	3 (< 1%)	2 (< 1%)	1 (< 1%)	5 (< 1%)	5 (< 1%)	0 (–)
Endocrinological	2 (< 1%)	1 (< 1%)	1 (< 1%)	0 (–)	0 (–)	0 (–)
Gastrointestinal	77 (8%)	49 (9%)	28 (8%)	227 (6%)	134 (6%)	93 (6%)
Genitourinary	7 (1%)	5 (1%)	2 (1%)	27 (1%)	22 (1%)	5 (< 1%)
Musculoskeletal	7 (1%)	5 (1%)	2 (1%)	43 (1%)	28 (1%)	12 (1%)
Neurological	16 (2%)	14 (2%)	2 (1%)	23 (1%)	19 (1%)	1 (< 1%)
Otological	4 (< 1%)	3 (1%)	1 (< 1%)	9 (< 1%)	7 (< 1%)	2 (< 1%)
Psychiatric/psychological	10 (1%)	8 (1%)	2 (1%)	73 (2%)	20 (1%)	46 (3%)
Respiratory	721 (78%)	438 (77%)	283 (80%)	3353 (83%)	2031 (84%)	1312 (83%)
Multiple systems	23 (2%)	14 (2%)	9 (3%)	92 (2%)	58 (2%)	34 (2%)
Unknown or not specified	34 (4%)	18 (3%)	16 (5%)	100 (2%)	42 (2%)	55 (3%)

### Pain Prevalence

The weekly prevalence of pain was 44% (mild 34%, moderate 9%, severe 1%). Pain prevalence was higher in males than in females (48% (mild 37%, moderate 9%, severe 2%) vs 41% (mild 30%, moderate 10%, severe 1%); PRR 1.18, 95% CI 1.0–1.3). During weeks when athletes reported pain, 28% of weeks involved concurrent injury (females 31%, males 26%), 8% involved concurrent illness (females 11%, males 6%), while for 65% of weeks no concurrent health problem was registered (females 59%, males 69%).

## Discussion

This study provides novel insights into injury, illness, and pain patterns among Olympic athletes. On average, one in four athletes reported a health problem in any given week, and each athlete incurred 5 health problems and 22 time-loss days per year. Injuries had higher prevalence than illnesses, but illnesses had higher incidence and imposed greater burden, particularly among female and winter sport athletes. Almost half of athletes reported sports-related pain during an average week.

### Injury Epidemiology

The prevalence, incidence, and burden of injury were lower in our cohort than among Norwegian Olympic athletes using comparable health surveillance [6]. This may partly be explained by differences in the study periods, as our data were collected continuously over three years, while the Norwegian data covered the 12–18 months preceding each Olympic Games. Differences in included sports may also contribute to the disparity; for instance, injury-prone sports such as handball and ice hockey were not represented in our cohort. Our injury incidence was similar to UK Olympic athletes based on medical records [8], but about three times higher than previously reported in Swedish insurance registry data [7]. This indicates that weekly self-reporting captures more injuries, particularly overuse injuries that may be underreported in insurance registries. We observed no differences in injury incidence or prevalence between sexes or competitive seasons, consistent with data from the Olympic Games [5] and from Swedish Olympic athletes [7].

### Illness Epidemiology

The prevalence and incidence of illnesses in our cohort were comparable to Norwegian Olympic athlete data [6], although the overall burden was greater in our cohort. We observed both illness incidence and burden greater than data from UK Olympic athletes, likely explained by their population being limited to summer sports [8]. Female and winter sport athletes were particularly affected by illness, similar to findings from other Olympic cohorts [5, 6].

Respiratory illnesses dominated, comprising 78% of all illness cases in our cohort. Potential respiratory illness risk factors include increased training load and monotony, lack of tapering, endurance training, competition periods, winter and altitude training, international travel, and vitamin D deficiency [19]. Thus, the high respiratory illness rates in winter sport athletes may be explained by increased pathogen exposure in winter, intense competition during the winter season, cold exposure, and frequent international travel [19].

### Pain Prevalence

Pain prevalence was high, and in almost two-thirds of weeks with reported pain no concurrent health problem was reported. Such ‘pain-only’ weeks may represent early-stage conditions (‘niggles’) not yet considered a reportable health problem. Previous research in football has shown that the risk of time-loss injury is increased when preceded by a self-reported complaint [20]. Pain, even without limitations to participation, may indicate underlying conditions that warrant further monitoring.

In elite sport, cultural norms may normalise pain and contribute to underreporting and delayed recognition of health problems. Athletes may distinguish ‘normal’ pain from pain considered a health problem, for example, when it limits performance or requires medical attention. Given that performance often is the main goal for elite athletes [21], they may accept the presence of pain to continue training and competing.

### Strengths and Limitations

This study has several strengths. First, it used a prospective design with year-round surveillance over three years, allowing a comprehensive overview of injury, illness, and pain patterns among Olympic athletes. Second, health problems were collected through systematic weekly surveillance using athlete self-reports via the OSTRC-H2, ensuring standardised collection of health problems, and follow-up of all health problems by SOC medical staff, improving quality and robustness of data.

Some limitations should be acknowledged. Self-reported data facilitate large-scale health surveillance but rely on athletes’ interpretation of injury or illness. Thus, misclassification or underreporting cannot be excluded. Several factors may influence athlete reporting in surveillance studies, and missing weekly responses may therefore not have been random. For example, athletes may have been less likely to complete the weekly questionnaire during periods of heavy training, competition, or travel, or because of perceived consequences of reporting, confidentiality concerns, and reporting fatigue over time (particularly among athletes with persistent or recurrent health problems). This may have led to underreporting and, consequently, underestimation of prevalence and incidence estimates in this study. Consecutive inclusion of athletes led to variation in observation periods and may have influenced estimates in smaller subgroups. For confidentiality reasons, sport-specific data were not reported, and sports were grouped into broader categories. These categories were used by the SOC high performance team, ensuring practical relevance, but no consensus exists on categorisation of Olympic sports [6–8]. Pain was captured only using question four in the OSTRC-O2 and analysed descriptively; therefore, we did not assess whether pain predicted subsequent injury or illness events. Despite follow-up of all health problems by SOC medical staff, a relatively high proportion of health problems were assigned a non-specific injury type. This likely reflects organisational constraints, including delayed or limited opportunities for in-person medical consultations, which may have reduced diagnostic precision for some health problems. Finally, we lacked medical staff-defined return to sport dates, limiting detailed analyses of time-loss and return to sport. Generalisability of the findings is limited, as injury and illness patterns may differ in other athlete populations.

### Clinical Implications

Athletes appear to continue participation despite injury, whereas illness typically results in complete but short-term time-loss. Consistent with other Olympic cohorts [5–8], knee, lumbosacral, shoulder, and ankle injuries were the most common. Injury prevention should prioritise these areas; for example, through targeted injury prevention exercise programmes. Such programmes have shown reductions in lower extremity and shoulder injuries in football and handball [22–25]. However, most evidence derives from lower-level team sports, and its effectiveness among Olympic athletes or individual sports remains uncertain. Illness prevention should prioritise respiratory illness and address modifiable risk factors. Strategies may include hygiene practice education, strict hygiene routines during travel, careful monitoring and adjustment of training load, and increased awareness of illness risk factors [26].

Pain was frequently reported without a concurrent health problem. Integrating pain surveillance alongside injury and illness surveillance may facilitate detection of early-stage conditions and timely initiation of rehabilitation or prevention strategies. Future research should explore pain as a potential risk factor for subsequent health problems and clarify how persistent pain influences performance and long-term athlete health.

Variation in prevalence, incidence, and burden across sexes, competitive seasons, and sport categories highlights the need for tailored prevention strategies. These findings can guide National Olympic Committees in prioritising prevention and care strategies most likely to improve athlete health and performance, while future research into risk factors may further inform targeted interventions to reduce the burden of health problems in Olympic athletes.

## Conclusion

One in four Swedish Olympic athletes reported a health problem in an average week, with 4.6 new health problems and 22 time-loss days per athlete per year. Injury burden was highest in winter sport athletes, and illness burden highest among females and winter sport athletes. The knee, lumbosacral region, shoulder, and ankle were the most common injury locations, and respiratory illness was the most common type of illness. Injury and illness patterns varied between subgroups, emphasising the need for tailored prevention and management strategies. Almost half of athletes reported sports-related pain during an average week, the significance of which should be explored further.

## Supplementary Information

Supplementary material 1.

Supplementary material 2.

Supplementary material 3.

Supplementary material 4.

Supplementary material 5.

## Data Availability

Data are available upon reasonable request.
