# Supplementary material for: Injury and Illness Prevalence and Incidence in Swedish Olympic Athletes: A 3-year Prospective Cohort Study
Source: Sports Med Open. 2026 Jun 3;12:62. doi: 10.1186/s40798-026-01035-8 (PMC13234072; doi:10.1186/s40798-026-01035-8)
Supplement: Supplementary file 1 — Supplementary material 1. [file 40798_2026_1035_MOESM1_ESM.pdf]

Title: Injury and illness prevalence and incidence in Swedish Olympic athletes: a 3-year prospective cohort study

Journal: Sports Medicine - Open

Authors: Kalle Torvaldsson [1, 2], Sofi Sonesson [1, 2], Hanna Lindblom [1, 2], Jörgen Sandberg [3], Lykke Tamm [3], Martin Hägglund [1, 2, 3]

Affiliations:

[1] Department of Health, Medicine and Caring Sciences, Unit of Physiotherapy, Linköping University, Linköping, Sweden

[2] Sport Without Injury ProgrammE (SWIPE), Department of Health, Medicine and Caring Sciences, Linköping University, Linköping, Sweden

[3] Swedish Olympic Committee, Sofiatornet, Olympiastadion, Stockholm, Sweden

Corresponding author: Kalle Torvaldsson (kalle.torvaldsson@liu.se)

Online Resource 1 Number of included athletes by competitive season, sport category, and sport.

Sport	Total	Female	Male
**Summer sports**	**144**	**73**	**71**
**Cyclic sports**	**41**	**17**	**24**
Athletics (middle-distance, long-distance, race walking)	3	1	2
Canoe	11	4	7
Cycling	4	2	2
Modern pentathlon	3	1	2
Rowing	5	1	4
Sport climbing	1	0	1
Swimming	11	7	4
Triathlon	3	1	2
**Full-contact sports**	**18**	**9**	**9**
Boxing	3	2	1
Judo	5	3	2
Wrestling	10	4	6
**High-impact sports**	**59**	**39**	**20**
Athletics (sprint, throwing, jumping, combined events)	17	10	7
Diving	3	3	0
Equestrian	22	18	4
Gymnastics	3	1	2
Sailing	10	7	3
Skateboarding	3	0	3
Weightlifting	1	0	1
**Precision sports**	**13**	**5**	**8**
Golf	3	2	1
Shooting	10	3	7
**Reactive sports**	**13**	**3**	**10**
Badminton	3	1	2
Beach volleyball	4	0	4
Fencing	1	1	0
Table tennis	4	0	4
Tennis	1	1	0
**Winter sports**	**81**	**38**	**43**
**Cyclic sports**	**30**	**17**	**13**
Biathlon	9	6	3
Cross-country skiing	14	8	6
Ski mountaineering	1	1	0
Speed skating	6	2	4
**High-impact sports**	**36**	**11**	**25**
Alpine skiing	9	7	2
Figure skating	1	0	1
Freestyle skiing	21	2	19
Luge	2	1	1
Ski jumping	1	1	0
Snowboarding	2	0	2
**Precision sports**	**15**	**10**	**5**
Curling	15	10	5
**Total**	**225**	**111**	**114**
