# Supplementary material for: Injury and Illness Prevalence and Incidence in Swedish Olympic Athletes: A 3-year Prospective Cohort Study
Source: Sports Med Open. 2026 Jun 3;12:62. doi: 10.1186/s40798-026-01035-8 (PMC13234072; doi:10.1186/s40798-026-01035-8)
Supplement: Supplementary file 2 — Supplementary material 2. [file 40798_2026_1035_MOESM2_ESM.pdf]

Title: Injury and illness prevalence and incidence in Swedish Olympic athletes: a 3-year prospective cohort study

Journal: Sports Medicine - Open

Authors: Kalle Torvaldsson [1, 2], Sofi Sonesson [1, 2], Hanna Lindblom [1, 2], Jörgen Sandberg [3], Lykke Tamm [3], Martin Hägglund [1, 2, 3]

Affiliations:
[1] Department of Health, Medicine and Caring Sciences, Unit of Physiotherapy, Linköping University, Linköping, Sweden
[2] Sport Without Injury ProgrammE (SWIPE), Department of Health, Medicine and Caring Sciences, Linköping University, Linköping, Sweden
[3] Swedish Olympic Committee, Sofiatornet, Olympiastadion, Stockholm, Sweden

Corresponding author: Kalle Torvaldsson (kalle.torvaldsson@liu.se)

Online Resource 2 Weekly prevalence and annual incidence by competitive season and sport category, for the total cohort.

	**Weekly prevalence (%, 95% CI)**	**Incidence (cases/athlete/year, 95% CI)**
HEALTH PROBLEMS		
**Summer sports**	**21.9 (19.5–24.5)**	**4.3 (3.9–4.8)**
Cyclic sports	21.2 (17.7–25.4)	5.1 (4.4–5.9)
Full-contact sports	31.7 (24.6–40.8)	6.8 (5.4–8.5)
High-impact sports	22.2 (18.1–27.3)	3.8 (3.2–4.5)
Precision sports	18.5 (13.0–26.4)	3.5 (2.5–4.8)
Reactive sports	16.5 (10.0–27.1)	2.1 (1.5–2.8)
**Winter sports**	**26.7 (23.5–30.4)**	**5.1 (4.5–5.7)**
Cyclic sports	24.9 (20.6–30.2)	6.9 (5.8–8.2)
High-impact sports	23.5 (18.5–29.7)	3.5 (2.9–4.2)
Precision sports	33.5 (27.3–41.1)	6.5 (5.4–7.9)
INJURIES		
**Summer sports**	**14.3 (12.2–16.9)**	**1.6 (1.3–1.8)**
Cyclic sports	11.3 (8.4–15.1)	1.4 (1.1–1.8)
Full-contact sports	21.4 (15.0–30.6)	2.7 (2.0–3.6)
High-impact sports	16.2 (12.3–21.4)	1.7 (1.3–2.2)
Precision sports	11.0 (6.5–18.7)	1.1 (0.7–1.7)
Reactive sports	14.0 (7.8–25.2)	1.0 (0.6–1.7)
**Winter sports**	**16.2 (13.0–20.1)**	**1.4 (1.2–1.8)**
Cyclic sports	10.4 (7.1–15.2)	2.1 (1.5–2.9)
High-impact sports	17.3 (12.5–24.1)	1.2 (0.9–1.7)
Precision sports	18.2 (12.3–26.9)	1.4 (0.9–2.0)
ILLNESSES		
**Summer sports**	**7.9 (7.0–8.9)**	**2.8 (2.5–3.1)**
Cyclic sports	10.5 (8.9–12.3)	3.7 (3.2–4.3)
Full-contact sports	11.4 (8.0–16.1)	4.1 (3.1–5.5)
High-impact sports	6.3 (5.0–7.9)	2.2 (1.7–2.7)
Precision sports	7.8 (5.3–11.5)	2.4 (1.6–3.6)
Reactive sports	2.5 (1.6–3.8)	1.1 (0.7–1.7)
**Winter sports**	**11.4 (9.9–13.1)**	**3.7 (3.3–4.2)**
Cyclic sports	14.8 (12.0–18.2)	4.9 (4.0–6.0)
High-impact sports	6.3 (5.0–8.0)	2.4 (1.9–2.9)
Precision sports	17.6 (14.4–21.6)	5.2 (4.2–6.3)
CI confidence interval		
