# Supplementary material for: Injury and Illness Prevalence and Incidence in Swedish Olympic Athletes: A 3-year Prospective Cohort Study
Source: Sports Med Open. 2026 Jun 3;12:62. doi: 10.1186/s40798-026-01035-8 (PMC13234072; doi:10.1186/s40798-026-01035-8)

Title: Injury and illness prevalence and incidence in Swedish Olympic athletes: a 3-year prospective cohort study

Journal: Sports Medicine - Open

Authors: Kalle Torvaldsson [1, 2], Sofi Sonesson [1, 2], Hanna Lindblom [1, 2], Jörgen Sandberg [3], Lykke Tamm [3], Martin Hägglund [1, 2, 3]

Affiliations:
[1] Department of Health, Medicine and Caring Sciences, Unit of Physiotherapy, Linköping University, Linköping, Sweden
[2] Sport Without Injury ProgrammE (SWIPE), Department of Health, Medicine and Caring Sciences, Linköping University, Linköping, Sweden
[3] Swedish Olympic Committee, Sofiatornet, Olympiastadion, Stockholm, Sweden

Corresponding author: Kalle Torvaldsson (kalle.torvaldsson@liu.se)

Online Resource 3 Weekly prevalence and annual incidence of injury and illness, stratified by age group. Numbers in brackets represent total athlete-weeks (prevalence) or athlete-years (incidence). Numbers above the bars represent 'any health problem'. Sums of injury and illness may exceed 'any health problem' when both an injury and an illness were reported in the same week.

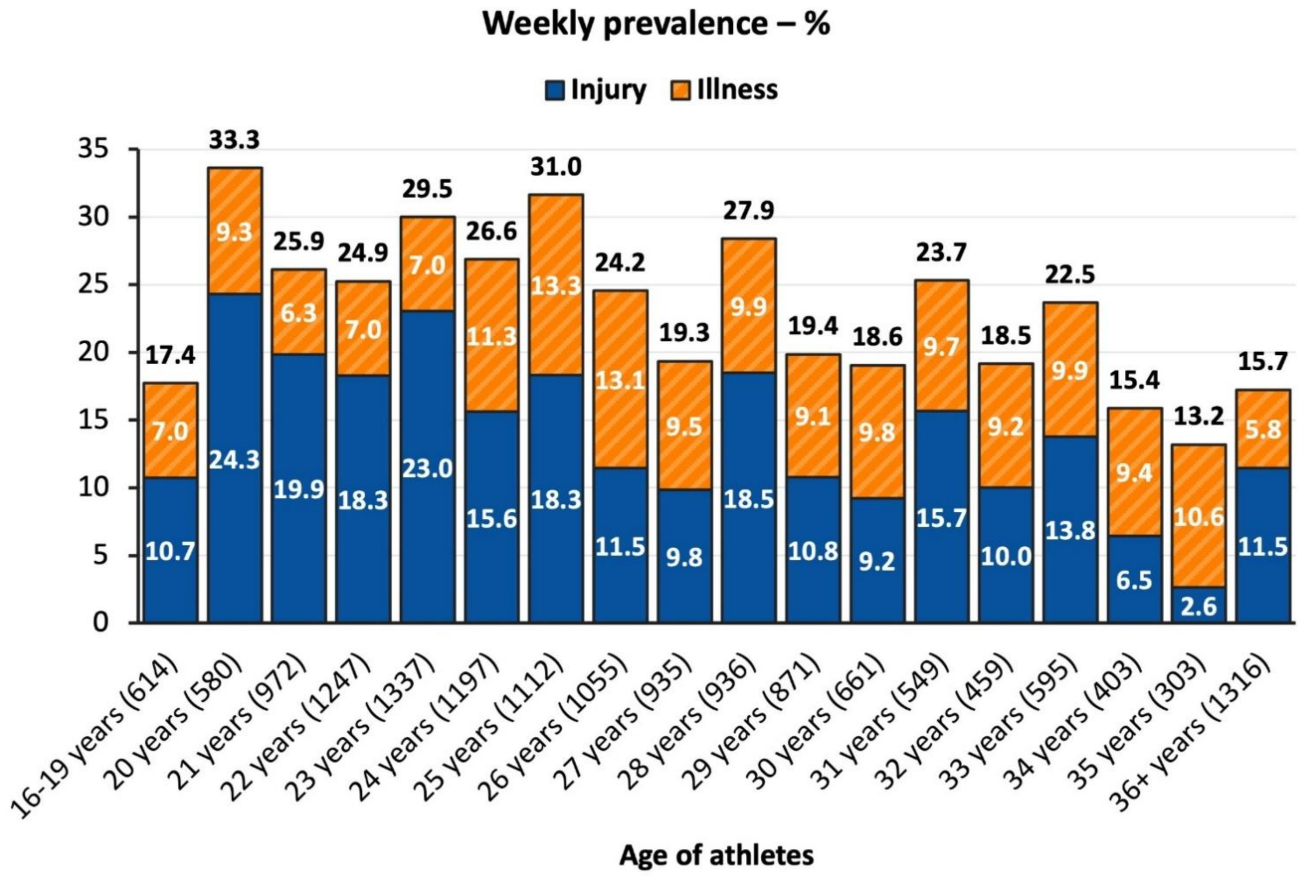

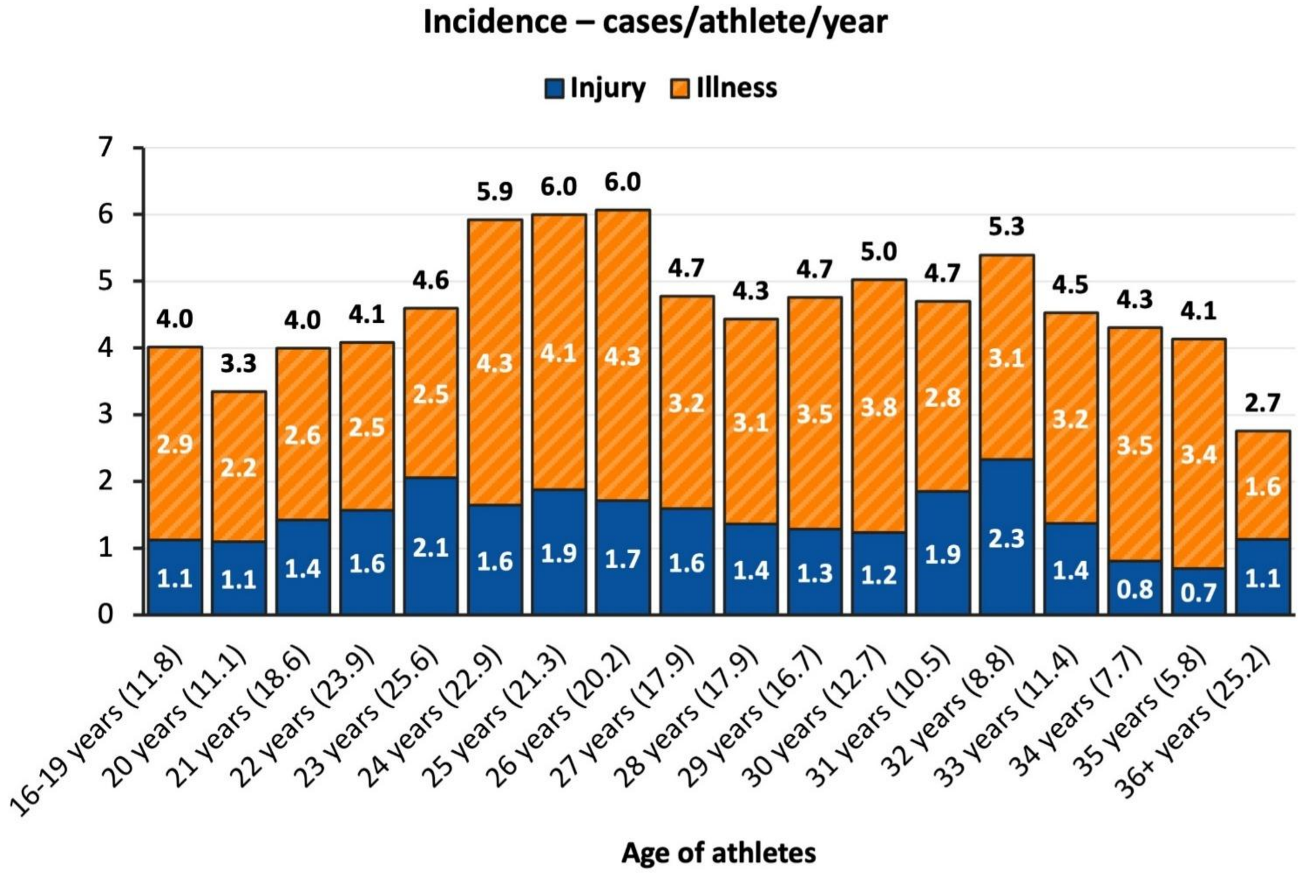

Supplement: Supplementary file 3 — Supplementary material 3. [file 40798_2026_1035_MOESM3_ESM.pdf]
