# Supplementary material for: Injury and Illness Prevalence and Incidence in Swedish Olympic Athletes: A 3-year Prospective Cohort Study
Source: Sports Med Open. 2026 Jun 3;12:62. doi: 10.1186/s40798-026-01035-8 (PMC13234072; doi:10.1186/s40798-026-01035-8)
Supplement: Supplementary file 4 — Supplementary material 4. [file 40798_2026_1035_MOESM4_ESM.pdf]

Title: Injury and illness prevalence and incidence in Swedish Olympic athletes: a 3-year prospective cohort study

Journal: Sports Medicine - Open

Authors: Kalle Torvaldsson [1, 2], Sofi Sonesson [1, 2], Hanna Lindblom [1, 2], Jörgen Sandberg [3], Lykke Tamm [3], Martin Hägglund [1, 2, 3]

Affiliations:
[1] Department of Health, Medicine and Caring Sciences, Unit of Physiotherapy, Linköping University, Linköping, Sweden
[2] Sport Without Injury ProgrammE (SWIPE), Department of Health, Medicine and Caring Sciences, Linköping University, Linköping, Sweden
[3] Swedish Olympic Committee, Sofiatornet, Olympiastadion, Stockholm, Sweden

Corresponding author: Kalle Torvaldsson (kalle.torvaldsson@liu.se)

Online Resource 4 Weekly prevalence and annual incidence by injury location, type, and onset, stratified by sex.

	Weekly prevalence (%, 95% CI)			Incidence (injuries/athlete/year, 95% CI)		
	Total	Female	Male	Total	Female	Male
**Injury location**						
**Head and neck**	**0.92 (0.58–1.46)**	**1.00 (0.54–1.88)**	**0.83 (0.42–1.64)**	**0.13 (0.09–0.19)**	**0.16 (0.10–0.26)**	**0.09 (0.05–0.18)**
Head	0.28 (0.17–0.48)	0.38 (0.19–0.76)	0.18 (0.09–0.40)	0.06 (0.04–0.11)	0.06 (0.03–0.12)	0.06 (0.03–0.16)
Neck	0.63 (0.34–1.18)	0.62 (0.25–1.56)	0.65 (0.28–1.51)	0.06 (0.04–0.11)	0.10 (0.05–0.18)	0.03 (0.01–0.07)
**Upper limb**	**3.85 (2.98–4.97)**	**3.65 (2.55–5.21)**	**4.05 (2.82–5.82)**	**0.37 (0.30–0.46)**	**0.37 (0.28–0.49)**	**0.37 (0.26–0.52)**
Shoulder	1.94 (1.32–2.85)	1.88 (1.14–3.08)	2.01 (1.12–3.59)	0.14 (0.11–0.20)	0.16 (0.11–0.24)	0.13 (0.08–0.21)
Upper arm	0.01 (0.00–0.04)	0.03 (0.01–0.07)	NA	0.01 (0.00–0.02)	0.01 (0.01–0.04)	NA
Elbow	0.46 (0.28–0.76)	0.36 (0.16–0.81)	0.57 (0.30–1.06)	0.05 (0.03–0.08)	0.04 (0.02–0.07)	0.06 (0.02–0.13)
Forearm	0.09 (0.03–0.32)	0.11 (0.03–0.36)	0.08 (0.01–0.87)	0.01 (0.00–0.06)	0.01 (0.00–0.03)	0.01 (0.00–0.16)
Wrist	1.12 (0.69–1.82)	0.89 (0.46–1.70)	1.36 (0.69–2.67)	0.08 (0.05–0.12)	0.09 (0.06–0.15)	0.07 (0.03–0.15)
Hand	0.69 (0.45–1.04)	0.50 (0.26–0.96)	0.87 (0.51–1.49)	0.08 (0.05–0.13)	0.06 (0.03–0.13)	0.10 (0.06–0.17)
**Trunk**	**3.27 (2.43–4.40)**	**2.88 (1.94–4.28)**	**3.66 (2.38–5.62)**	**0.31 (0.25–0.39)**	**0.28 (0.20–0.39)**	**0.34 (0.25–0.45)**
Chest	0.17 (0.10–0.30)	0.15 (0.06–0.34)	0.20 (0.09–0.42)	0.03 (0.02–0.05)	0.03 (0.01–0.06)	0.04 (0.02–0.07)
Thoracic spine	0.15 (0.07–0.29)	0.21 (0.09–0.48)	0.08 (0.02–0.31)	0.04 (0.02–0.06)	0.05 (0.03–0.10)	0.02 (0.01–0.07)
Lumbosacral	2.81 (2.00–3.95)	2.48 (1.59–3.88)	3.14 (1.91–5.16)	0.20 (0.16–0.27)	0.19 (0.13–0.29)	0.22 (0.15–0.32)
Abdomen	0.15 (0.08–0.25)	0.05 (0.01–0.20)	0.24 (0.13–0.43)	0.04 (0.02–0.07)	0.01 (0.00–0.07)	0.06 (0.04–0.11)
**Lower limb**	**7.71 (6.30–9.42)**	**7.35 (5.63–9.59)**	**8.07 (5.99–10.87)**	**0.65 (0.54–0.78)**	**0.71 (0.55–0.91)**	**0.59 (0.45–0.78)**
Hip/groin	0.75 (0.47–1.19)	0.61 (0.33–1.13)	0.88 (0.46–1.71)	0.09 (0.06–0.14)	0.08 (0.04–0.16)	0.10 (0.06–0.16)
Thigh	0.59 (0.32–1.07)	0.45 (0.17–1.16)	0.73 (0.34–1.56)	0.07 (0.04–0.13)	0.07 (0.03–0.18)	0.08 (0.04–0.16)
Knee	3.91 (2.90–5.28)	4.61 (3.11–6.83)	3.21 (2.02–5.10)	0.23 (0.18–0.30)	0.25 (0.18–0.36)	0.21 (0.14–0.31)
Lower leg	0.30 (0.16–0.59)	0.57 (0.28–1.13)	0.04 (0.01–0.16)	0.05 (0.02–0.12)	0.08 (0.03–0.21)	0.02 (0.01–0.09)
Ankle	1.89 (1.18–3.03)	0.75 (0.40–1.42)	3.02 (1.71–5.34)	0.10 (0.06–0.15)	0.09 (0.04–0.19)	0.10 (0.06–0.17)
Foot	0.66 (0.43–1.01)	0.71 (0.38–1.32)	0.61 (0.34–1.09)	0.11 (0.07–0.15)	0.12 (0.07–0.19)	0.09 (0.05–0.16)
**Multiple regions**	**0.53 (0.28–1.01)**	**0.52 (0.22–1.19)**	**0.55 (0.21–1.43)**	**0.06 (0.04–0.08)**	**0.06 (0.04–0.11)**	**0.05 (0.03–0.09)**
**Injury type**						
**Muscle/tendon**	**3.94 (3.05–5.08)**	**4.37 (3.11–6.16)**	**3.50 (2.39–5.13)**	**0.43 (0.35–0.53)**	**0.49 (0.38–0.65)**	**0.38 (0.27–0.51)**
Muscle injury	1.33 (0.93–1.91)	1.48 (0.89–2.47)	1.19 (0.72–1.95)	0.22 (0.17–0.29)	0.23 (0.16–0.34)	0.21 (0.14–0.30)
Muscle contusion	0.32 (0.16–0.63)	0.52 (0.23–1.16)	0.12 (0.05–0.29)	0.06 (0.04–0.10)	0.09 (0.05–0.16)	0.04 (0.01–0.09)
Muscle compartment syndrome	0.08 (0.03–0.19)	0.09 (0.03–0.30)	0.07 (0.02–0.22)	0.01 (0.00–0.05)	0.01 (0.00–0.03)	0.01 (0.00–0.12)
Tendinopathy	1.89 (1.28–2.78)	2.18 (1.30–3.66)	1.60 (0.90–2.85)	0.13 (0.09–0.19)	0.15 (0.09–0.24)	0.12 (0.07–0.20)
Tendon rupture	0.38 (0.13–1.16)	0.24 (0.07–0.81)	0.53 (0.12–2.40)	0.01 (0.00–0.02)	0.01 (0.01–0.04)	NA
**Nervous**	**0.38 (0.15–0.97)**	**0.37 (0.09–1.49)**	**0.38 (0.11–1.37)**	**0.03 (0.01–0.07)**	**0.03 (0.01–0.07)**	**0.03 (0.01–0.12)**
Brain/spinal cord injury / Peripheral nerve injury	0.38 (0.15–0.97)	0.37 (0.09–1.49)	0.38 (0.11–1.37)	0.03 (0.01–0.07)	0.03 (0.01–0.07)	0.03 (0.01–0.12)
**Bone**	**2.25 (1.47–3.43)**	**1.06 (0.50–2.23)**	**3.43 (2.07–5.68)**	**0.11 (0.08–0.15)**	**0.08 (0.04–0.15)**	**0.14 (0.10–0.21)**
Fracture	1.78 (1.07–2.96)	0.66 (0.22–2.00)	2.89 (1.63–5.13)	0.06 (0.04–0.10)	0.03 (0.01–0.11)	0.09 (0.06–0.15)
Bone stress injury	0.24 (0.08–0.72)	0.09 (0.02–0.44)	0.40 (0.11–1.42)	0.01 (0.01–0.04)	0.01 (0.00–0.13)	0.02 (0.01–0.05)
Bone contusion	0.22 (0.11–0.45)	0.30 (0.13–0.74)	0.15 (0.05–0.41)	0.04 (0.02–0.06)	0.04 (0.02–0.09)	0.03 (0.01–0.07)
**Cartilage/synovium/bursa**	**2.11 (1.43–3.12)**	**1.41 (0.94–2.14)**	**2.81 (1.62–4.87)**	**0.11 (0.08–0.17)**	**0.10 (0.06–0.17)**	**0.13 (0.07–0.22)**
Cartilage	1.43 (0.87–2.36)	0.98 (0.59–1.62)	1.89 (0.93–3.85)	0.07 (0.04–0.12)	0.08 (0.04–0.14)	0.06 (0.03–0.15)
Arthritis	0.21 (0.08–0.54)	0.18 (0.07–0.50)	0.24 (0.05–1.06)	0.01 (0.00–0.03)	0.01 (0.00–0.05)	NA
Synovitis/capsulitis	0.21 (0.08–0.54)	0.24 (0.07–0.76)	0.18 (0.04–0.86)	0.01 (0.00–0.04)	0.01 (0.00–0.12)	0.01 (0.00–0.05)
Bursitis	0.68 (0.31–1.50)	0.01 (0.00–0.30)	1.35 (0.61–3.00)	0.02 (0.01–0.05)	NA	0.05 (0.03–0.09)
**Ligament/joint capsule**	**2.44 (1.71–3.48)**	**3.37 (2.20–5.15)**	**1.52 (0.80–2.87)**	**0.17 (0.12–0.25)**	**0.20 (0.13–0.31)**	**0.14 (0.08–0.26)**
Joint sprain (ligament tear or acute instability episode)	2.03 (1.38–2.97)	2.54 (1.57–4.09)	1.52 (0.80–2.87)	0.16 (0.11–0.23)	0.18 (0.12–0.29)	0.14 (0.08–0.26)
Chronic instability	0.43 (0.18–1.05)	0.86 (0.35–2.09)	NA	0.01 (0.00–0.03)	0.02 (0.01–0.07)	NA
**Superficial tissues/skin / Vessels**	**0.38 (0.22–0.63)**	**0.38 (0.22–0.66)**	**0.37 (0.15–0.90)**	**0.08 (0.05–0.12)**	**0.08 (0.05–0.15)**	**0.07 (0.03–0.15)**
Contusion (superficial) / Vascular trauma	0.26 (0.13–0.50)	0.25 (0.14–0.46)	0.26 (0.08–0.83)	0.05 (0.03–0.08)	0.06 (0.04–0.11)	0.04 (0.02–0.08)
Laceration	0.11 (0.04–0.26)	0.13 (0.04–0.40)	0.08 (0.02–0.37)	0.02 (0.01–0.06)	0.02 (0.01–0.09)	0.02 (0.00–0.09)
Abrasion	0.01 (0.00–0.09)	NA	0.03 (0.00–0.18)	0.01 (0.00–0.05)	NA	0.01 (0.00–0.10)
**Non-specific**	**4.56 (3.66–5.68)**	**4.35 (3.17–5.95)**	**4.77 (3.50–6.49)**	**0.58 (0.49–0.69)**	**0.60 (0.47–0.77)**	**0.56 (0.43–0.72)**
**Injury onset**						
Gradual onset	7.72 (6.47–9.21)	7.91 (6.19–10.11)	7.53 (5.84–9.71)	0.78 (0.67–0.92)	0.80 (0.65–1.00)	0.76 (0.60–0.96)
Sudden onset	7.59 (6.19–9.30)	6.99 (5.31–9.20)	8.19 (6.09–11.00)	0.73 (0.62–0.87)	0.78 (0.62–0.99)	0.69 (0.54–0.88)

CI confidence interval, *NA* not applicable (e.g., due to no injury cases or small sample)
