# Supplementary material for: Injury and Illness Prevalence and Incidence in Swedish Olympic Athletes: A 3-year Prospective Cohort Study
Source: Sports Med Open. 2026 Jun 3;12:62. doi: 10.1186/s40798-026-01035-8 (PMC13234072; doi:10.1186/s40798-026-01035-8)
Supplement: Supplementary file 5 — Supplementary material 5. [file 40798_2026_1035_MOESM5_ESM.pdf]

Title: Injury and illness prevalence and incidence in Swedish Olympic athletes: a 3-year prospective cohort study

Journal: Sports Medicine - Open

Authors: Kalle Torvaldsson [1, 2], Sofi Sonesson [1, 2], Hanna Lindblom [1, 2], Jörgen Sandberg [3], Lykke Tamm [3], Martin Hägglund [1, 2, 3]

Affiliations:

[1] Department of Health, Medicine and Caring Sciences, Unit of Physiotherapy, Linköping University, Linköping, Sweden

[2] Sport Without Injury ProgrammE (SWIPE), Department of Health, Medicine and Caring Sciences, Linköping University, Linköping, Sweden

[3] Swedish Olympic Committee, Sofiatornet, Olympiastadion, Stockholm, Sweden

Corresponding author: Kalle Torvaldsson (kalle.torvaldsson@liu.se)

Online Resource 5 Weekly prevalence and annual incidence by illness organ system/region, stratified by sex.

	Weekly prevalence (%, 95% CI)			Incidence (illnesses/athlete/year, 95% CI)		
	**Total**	**Female**	**Male**	**Total**	**Female**	**Male**
**Organ system/region**						
Cardiovascular	0.01 (0.00–0.94)	NA	0.01 (0.00–1.92)	NA	NA	NA
Dermatological	0.18 (0.09–0.37)	0.29 (0.13–0.67)	0.08 (0.03–0.21)	0.07 (0.04–0.12)	0.10 (0.05–0.20)	0.03 (0.01–0.10)
Dental	0.03 (0.01–0.09)	0.04 (0.01–0.18)	0.01 (0.00–0.05)	0.01 (0.00–0.03)	0.01 (0.00–0.05)	0.01 (0.00–0.03)
Endocrinological	0.01 (0.00–0.10)	0.01 (0.00–0.05)	0.01 (0.00–0.57)	0.01 (0.00–0.05)	0.01 (0.00–0.03)	0.01 (0.00–0.30)
Gastrointestinal	0.70 (0.51–0.97)	0.94 (0.60–1.47)	0.46 (0.33–0.64)	0.26 (0.21–0.32)	0.33 (0.25–0.43)	0.19 (0.14–0.27)
Genitourinary	0.06 (0.03–0.14)	0.09 (0.04–0.23)	0.03 (0.00–0.18)	0.02 (0.01–0.06)	0.03 (0.01–0.08)	0.01 (0.00–0.10)
Musculoskeletal	0.12 (0.03–0.48)	0.16 (0.05–0.55)	0.08 (0.00–2.39)	0.02 (0.01–0.06)	0.03 (0.01–0.11)	0.01 (0.00–0.07)
Neurological	0.13 (0.08–0.21)	0.24 (0.14–0.40)	0.03 (0.01–0.08)	0.06 (0.03–0.09)	0.10 (0.06–0.16)	0.01 (0.00–0.04)
Otological	0.04 (0.01–0.10)	0.07 (0.02–0.19)	0.01 (0.00–0.13)	0.01 (0.01–0.04)	0.02 (0.01–0.06)	0.01 (0.00–0.07)
Psychiatric/psychological	0.15 (0.06–0.33)	0.13 (0.05–0.35)	0.16 (0.04–0.56)	0.03 (0.02–0.08)	0.06 (0.02–0.15)	0.01 (0.01–0.04)
Respiratory	7.32 (6.62–8.08)	8.99 (7.97–10.13)	5.65 (4.78–6.68)	2.43 (2.22–2.66)	2.94 (2.63–3.29)	1.92 (1.66–2.23)
Multiple systems	0.20 (0.13–0.32)	0.26 (0.16–0.45)	0.15 (0.06–0.35)	0.08 (0.05–0.12)	0.10 (0.06–0.16)	0.06 (0.03–0.15)
Unknown or not specified	0.28 (0.19–0.40)	0.29 (0.18–0.48)	0.26 (0.15–0.46)	0.11 (0.08–0.16)	0.12 (0.08–0.20)	0.10 (0.06–0.17)

CI confidence interval, *NA* not applicable (e.g., due to no illness cases or small sample)
